# Machine learning workflows detect ground-dwelling arthropods against heterogenous backgrounds

**DOI:** 10.1093/jisesa/ieag091

**Published:** 2026-09-01

**Authors:** Robert J Boyd, Kayla I Perry, Thomas P Franzem, Christie Bahlai

**Affiliations:** Department of Biological Sciences, Kent State University, Kent, OH, USA; Department of Entomology, The Ohio State University, Wooster, OH, USA; Department of Biological Sciences, Kent State University, Kent, OH, USA; Department of Biological Sciences, Kent State University, Kent, OH, USA; Kent State University Environmental Science and Design Research Institute, Kent, OH, USA

**Keywords:** camera monitoring, ground-dwelling arthropods, object detection

## Abstract

Camera-based monitoring of arthropods is an emerging tool for studying activity, abundance, and behavior. However, many current approaches rely on custom-built camera systems and complex analytical workflows, which limit accessibility and broader adoption. In addition, most arthropod camera-monitoring systems image subjects against uniform backgrounds, conditions that may alter natural activity patterns and reduce ecological realism. Here, we present a camera-monitoring workflow designed to increase accessibility by using commercially available game cameras coupled with a streamlined image-processing pipeline. We evaluated the performance of this approach by imaging arthropods against a complex, heterogeneous background to assess its effectiveness under conditions more representative of field environments. Our object-detection model performed reliably across evaluation metrics and accurately identified arthropods captured in the images despite background complexity. These results demonstrate that low-cost, widely available camera systems can be integrated with simplified analytical workflows to enable effective arthropod monitoring without specialized hardware. This approach provides a scalable and accessible method for studying arthropods in more natural contexts and may facilitate broader adoption of camera-based monitoring in ecological research and biodiversity assessments.

## Graphical abstract

**Graphical Abstract:**
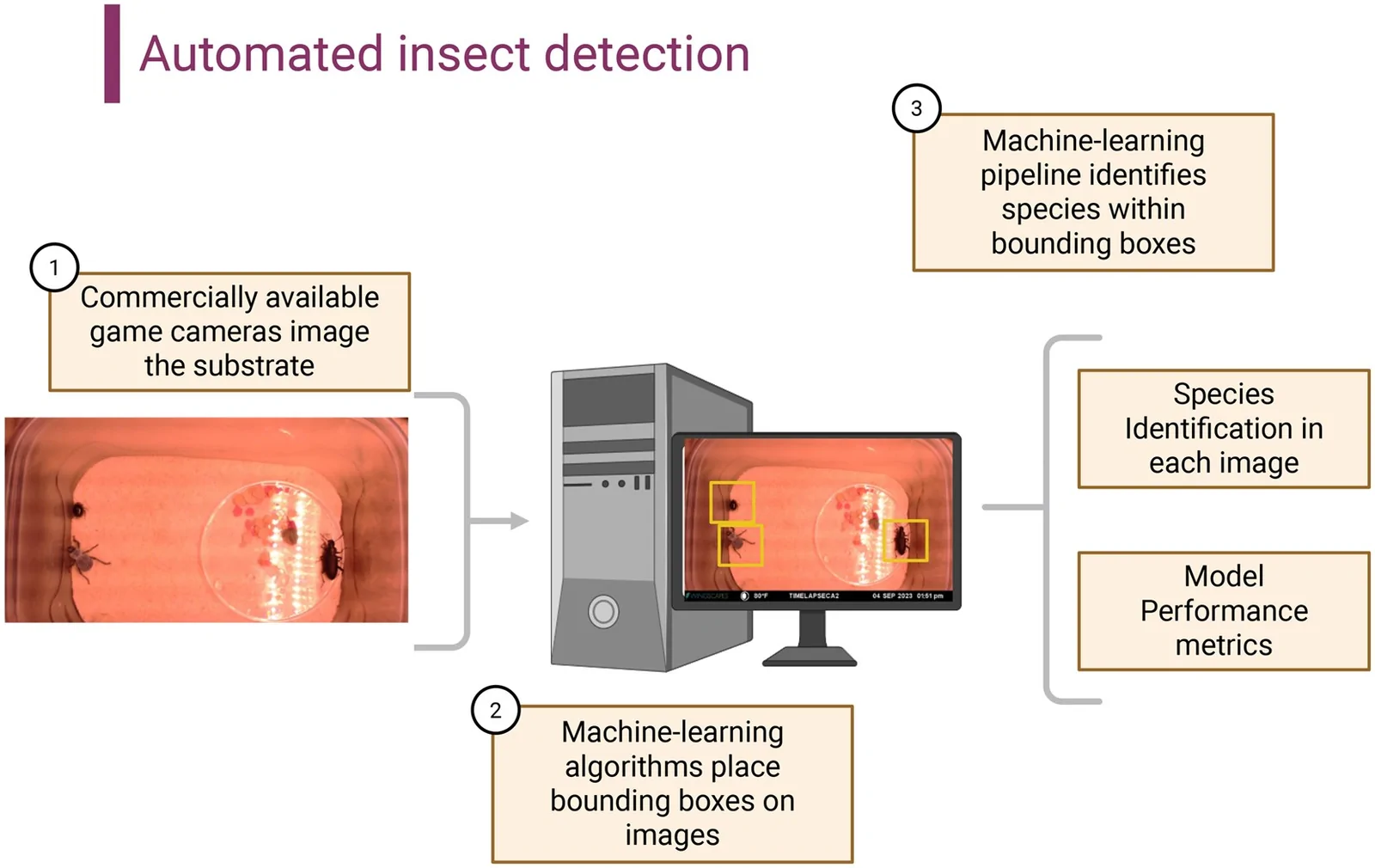

## Introduction

Monitoring insect communities is challenging (Van Klink et al. 2022). Insects are small, occur in heterogenous environments, and require taxonomic expertise to identify. Many traps exist to collect data on insect biodiversity. Traditional collection methods provide physical specimens and trait data from discrete sampling events (Nachman et al. 2023), but there is growing consensus in the entomological community that nonextractive sampling (ie sampling that does not remove physical specimens from the environment) should be prioritized (Fischer and Larson 2019) to mitigate harm to naturally occurring populations. Further, given recently observed declines in global insect diversity, richness, and abundance (Wagner 2020), there is a pressing need for more insect biodiversity data. However, there is a bottleneck in terms of the personnel and resources available to collect insect data. Thus, a semiautomated workflow that can produce continuous, high volumes of insect occurrence data would be highly valuable.

Camera trapping is an emerging nonextractive method that utilizes cameras to photograph insects (Høye et al. 2021). This approach can provide biodiversity data on insects as well as diel and seasonal behavior and activity patterns with minimal environmental impact (Burton et al. 2015, Norouzzadeh et al. 2018) and indeed can be extended to applications such as invasive species detection or behavior studies. Camera trapping may be especially useful in combination with limited traditional insect trapping by coupling voucher-based trait information with time-resolved activity measures. However, camera traps produce a high volume of images, which create challenges in processing data (Valan et al. 2019, Engel et al. 2021) and have limited the adoption of camera-based monitoring approaches (Bjerge et al. 2023). Recent advances in computer vision may help to automate image sorting and identification in camera-trap workflows (Roy et al. 2024). To unlock the potential of camera traps in insect biodiversity monitoring (East et al. 2025), an analytic pipeline that integrates model training and data processing in controlled settings is necessary to establish proof-of-concept and prepare tools for field deployment. Although camera trapping has been used across a wide variety of insect monitoring applications (Preti et al. 2021, Naqvi et al. 2022; Høye et al. 2025), the development of techniques involving living ground-dwelling organisms sampled in situ has been relatively limited, with the majority of these studies occurring in agricultural settings over protracted time periods (Seimandi-Corda et al. 2024, Seimandi‐Corda et al. 2025). Some studies focusing on ground-dwelling arthropods rely on destructive sampling to gain species-level identifications (Hoekman et al. 2017), increasing latency time from sampling to validated data. Our work is intended to form the basis of real-time monitoring that can be deployed by practitioners, is easily repeated, and thus can provide long-term insights of change in slow ecological processes (ie differential trajectories of differing methods of forest restoration).

Widespread adoption of these tools has been limited by steep learning curves for key software frameworks and a reliance on custom camera systems that are not always accessible or user-friendly (Abadi et al. 2016, Paszke et al. 2019, Bjerge et al. 2023). While some coding is still required, modern object-detection models such as Ultralytics YOLO (“You Only Look Once”) have made high performance object-detection models more accessible (Stark et al. 2023). Although these technologies are increasing in use, applications to insects have been limited. For example, most studies have focused on pollinators and pests (Lv et al. 2022, Venverloo and Duarte 2024), while ground-dwelling insect communities, which are ecologically important and taxonomically diverse, are understudied (Roy et al. 2024). On the hardware side, most camera traps use custom, low-cost Raspberry Pi camera modules (Sittinger et al. 2024, Wittmann et al. 2024), but these systems still require coding and 3D printing which limits accessibility. Few studies evaluate commercially available cameras which can be calibrated and deployed immediately (Alison et al. 2022). Approaches using off-the-shelf cameras and focusing on ground-dwelling arthropods could increase accessibility of these tools and expand their implementation.

Here, we assess the performance of commercially available game cameras to capture records of live ground-dwelling beetles in a lab setting against a complex background. We then develop an analytic workflow that processes images and identifies beetles in an object-detection model. To our knowledge there are no published works that evaluate camera hardware and machine learning approaches to monitor beetles engaging in typical movement patterns. Further, our workflow is modular in terms of hardware and software, thus enabling easy modification and adaptation by other researchers. Our goal is to provide baseline data that can guide hardware selection and workflow development for in-the-field insect biodiversity monitoring.

## Experimental Design

### Object Detection Approach

Object detection is a core task in computer vision that involves not only identifying whether an object of interest is present in an image but also locating it with bounding boxes, assigning class labels, and providing confidence scores (Szeliski 2022, Chapter 6). In recent years, single-stage detectors such as YOLO have become widely used because they unify this pipeline into a highly efficient architecture, enabling real-time detection without the 2-stage proposal step of previous object-detection models (Redmon et al. 2016). The YOLO family has continued to evolve, with Ultralytics’ YOLOv8 offering 5 model sizes (n, s, m, l, x), ranging from lightweight versions optimized for edge devices to the large YOLOv8x, which contains 68,154,534 parameters (Pereira 2024). Ultralytics provides clear documentation and open-source implementations, making these models accessible to practitioners. In ecology and entomology, smaller YOLO variants have been adopted for custom Raspberry Pi and trap-based setups where real-time detection is desirable (Sittinger et al. 2024, Huang et al. 2025).

For this study, we selected YOLOv8x for our processing pipeline. While it is the largest and most computationally intensive model in the YOLOv8 family, its higher representational capacity may better handle the complexity of future field deployments. In this study, we train YOLOv8x in a controlled laboratory environment to provide groundwork to later apply transfer learning for outdoor monitoring, where photographic conditions will be less controlled and samples will be more sparse. Transfer learning is especially useful in ecological applications where annotated datasets are often limited, as it allows models pretrained on large datasets to adapt to smaller, domain-specific ones. Studies in entomology have shown that transfer learning can significantly improve classification and detection accuracy under these conditions (Valan et al. 2019, Spiesman et al. 2021, Bjerge et al. 2024).

### Camera Selection

We used the Wingscapes TimelapseCam Pro (Wingscapes/PRADCO Outdoor Brands; Calera, Alabama, United States) because it is commercially available, can capture high-resolution images at a focal distance of <0.5 m, has flash capability for nighttime photography, and supports configurable time intervals for image capture independent of motion triggers. These features were considered critical for maintaining image quality under diverse conditions and ensuring that photographs would be compatible with YOLO-based object-detection workflows (Bjerge et al. 2023).

### Beetle Arena Setup

All photographs were collected in a controlled laboratory environment using 3 beetle species housed in 38-cm tall black plastic totes. The test subjects for this study include 3 species: the blue death feigning beetle, *Asbolus verrucosus* LeConte (Coleoptera: Tenebrionidae), a representative darkling beetle, *Tenebrio molitor* Linnaeus (Coleoptera: Tenebrionidae), and the hairy robot beetle, *Edrotes ventricosus* LeConte (Coleoptera: Tenebrionidae). These species were chosen due to their availability in the pet trade and their visual distinctiveness from each other. Each tote was fitted with a layer of sand covering the base and furnished with standardized habitat features to create a heterogenous environment: 2 pieces of artificial foliage positioned near the center, food and water dishes placed at opposite corners, and a small hollow resin aquarium/terrarium piece to provide the insects with shelter in another corner. To prevent escape of subject insects and to provide a stable platform for the Wingscapes TimelapseCam Pro, chicken-wire mesh was secured across the top of each tote. This arrangement provided both cover and feeding opportunities while ensuring consistency across replicates. Overhead grow lights were used to maintain uniform illumination and maintain consistent temperature across each arena.

### 10-s Arena Setup

**Fig. 1.**
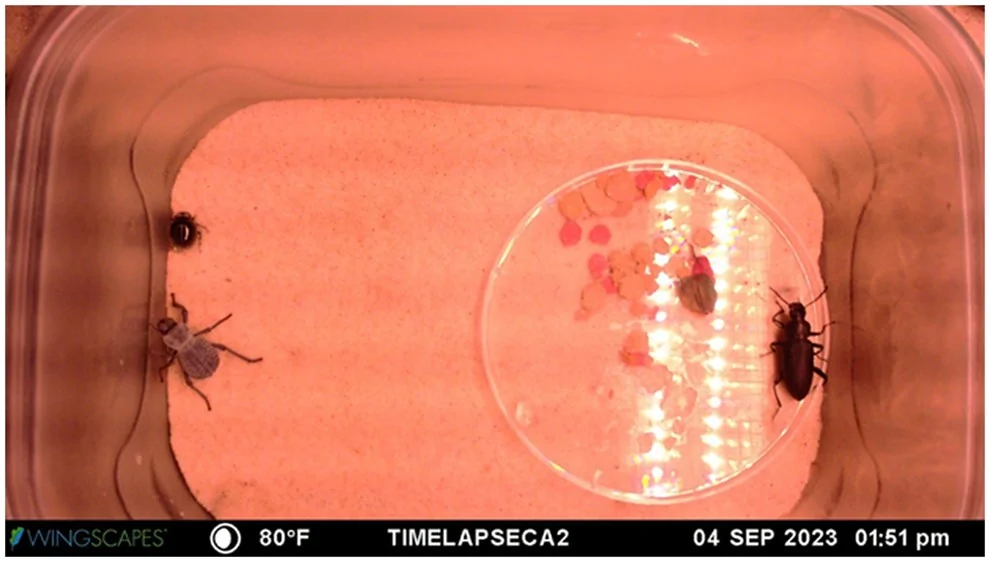
Calibration arena with camera mounted 38 cm above the tote bottom (10‑s calibration set in a smaller clear container). Right: darkling beetle (*Tenebrio molitor*). Bottom left: blue death‑feigning beetle (*Asbolus verrucosus*). Top left: hairy robot beetle (*Edrotes ventricosus*).

To create an initial dataset that ensured the majority of images taken would capture an insect, we created a smaller arena (Fig. 1) where the movement of insects was restricted and photos were taken at a high frequency (every 10 s). This approach was necessary to facilitate transfer learning for the first YOLO model and assist with the annotation of a larger dataset. To limit the movement of subjects, beetles were housed in clear, Ziploc-brand sandwich containers with a small amount of sand and a food/water dish placed inside of the larger tote arena. For each container, a random configuration of species was chosen from our colony stock, with the number of individuals per species ranging from 0 to 4 (Table 1). A camera unit was mounted above the enclosure and configured to capture images at maximum quality (6,080 × 3,420 pixels) at 10-s intervals. Each experimental set had a random duration, ranging from 62 min 10 s to 169 min 22 s.

**Table 1.** Images per species configuration for the calibration dataset

Configuration	Images per configuration
**Background images**	1,876
**1 blue, 1 hairy, 1 darkling**	889
**3 blue, 0 hairy, 0 darkling**	599
**1 blue, 0 hairy, 0 darkling**	515
**0 blue, 0 hairy, 1 darkling**	470
**1 blue, 0 hairy, 1 darkling**	468
**0 blue, 1 hairy, 1 darkling**	466
**0 blue, 1 hairy, 0 darkling**	454
**1 blue, 1 hairy, 0 darkling**	420
**0 blue, 0 hairy, 2 darkling**	406
**0 blue, 4 hairy, 0 darkling**	330

Each configuration included varying numbers of blue death feigning beetles (*Asbolus verrucosus*—“blue”), hairy robot beetles (*Edrotes ventricosus*—“hairy”), and darkling beetles (*Tenebrio molitor*—“darkling”).

To reduce early false positives, a mostly background_**‑**_only image set from standard tote run was added. Because this 10-s dataset was designed to bootstrap annotation and provide a base model for transfer learning, the 0 to 4 abundance grades (Table 1) represent training configurations rather than ecological replicates, and the setup was not intended for future density or abundance estimation; instead, we emphasized balancing the total number of insects per species to limit class bias.

### 1-min Arena Setup

The main dataset used the larger arenas and a 1-min sampling interval and was used to train a robust YOLO model capable of identifying insect species under variable conditions, including partial occlusion. The larger arenas were set within a 46 cm × 40 cm × 40 cm plastic totes. This dataset comprised 25 unique species configurations, each with the number of individuals per species ranging from 0 to 4 alone and in combination with individuals from other species (Table 2). Unlike the initial calibration dataset, insects were allowed to move freely within the totes. A camera unit was mounted above the enclosures to capture images at 1 min intervals at maximum quality (6,080 × 3,420 pixels).

**Table 2.** Images per species configuration for the 1-min dataset

Configuration	Images per configuration
**3 blue, 3 hairy, 3 darkling**	4,069
**0 blue, 3 hairy, 2 darkling**	3,375
**2 blue, 2 hairy, 2 darkling**	3,079
**0 blue, 0 hairy, 3 darkling**	2,980
**2 blue, 1 hairy, 4 darkling**	2,313
**3 blue, 4 hairy, 4 darkling**	2,211
**4 blue, 3 hairy, 0 darkling**	2,208
**1 blue, 1 hairy, 1 darkling**	1,876
**1 blue, 3 hairy, 3 darkling**	1,846
**2 blue, 4 hairy, 2 darkling**	1,836
**2 blue, 2 hairy, 4 darkling**	1,662
**3 blue, 2 hairy, 0 darkling**	1,662
**3 blue, 2 hairy, 3 darkling**	1,641
**3 blue, 4 hairy, 2 darkling**	1,620
**3 blue, 2 hairy, 1 darkling**	1,589
**0 blue, 0 hairy, 2 darkling**	1,562
**4 blue, 0 hairy, 4 darkling**	1,559
**1 blue, 4 hairy, 3 darkling**	1,552
**0 blue, 0 hairy, 4 darkling**	1,540
**1 blue, 4 hairy, 2 darkling**	1,458
**3 blue, 1 hairy, 1 darkling**	1,355
**0 blue, 3 hairy, 1 darkling**	1,233
**3 blue, 3 hairy, 2 darkling**	1,225
**3 blue, 4 hairy, 3 darkling**	945
**4 blue, 1 hairy, 0 darkling**	944
**4 blue, 0 hairy, 0 darkling**	720

Each configuration included varying numbers of blue death feigning beetles (*Asbolus verrucosus*—“blue”), hairy robot beetles (*Edrotes ventricosus*—“hairy”), and darkling beetles (*Tenebrio molitor*—“darkling”).

Image capture was limited to the hours between 08:00 and 21:00, with each experimental set lasting between 2 and 3 days. Temperatures were recorded throughout the trial, and insect health and presence were verified once per day. If insects were found deceased, the set would be restarted.

### Image Annotation

For the 10-s dataset, all images were annotated using Label Studio (Tkachenko et al. 2025) and LabelImg (Tkachenko et al. 2025). Bounding boxes were drawn around each beetle and labeled by species. Bounding boxes are rectangles drawn around each beetle, stored in YOLO format as class, x center, y center, width, and height. To ensure data quality, images were screened and removed if any portion of an insect was obscured by over 50%, including instances where individuals were occluded by other insects. Following this initial data cleaning, the annotated images were partitioned into an 80% training subset and a 20% validation subset for model development. We elected to utilize only training and validation data subsets rather than a training/validation/testing subsets because our experimental setup was based on controlled environments with only 3 insect species, thus there was very little sample variation. A test subset would draw from the same environment and essentially mirror the validation set, thus reducing the data available for the training subset. Although using only training and validation subsets could produce slightly inflated model performance metrics, given our experimental setup, we expect this was not an issue. Further, previous object-detection workflows aimed at identifying insects have used similar training/validation split ratios with no formal test subset (Zarboubi et al. 2026).

For the 1-min dataset, the YOLO model trained on the 10-s dataset was used to assist the image_**‑**_annotation process by running predictions. The prediction outputs were converted into YOLO text files (class, x center, y center, width, height) and loaded into Label Studio and LabelImg to speed the review for 40,060 images. In this case, no images were discarded due to insect visibility or due to lack of insect presence to ensure training data would contain null data and imperfect captures. As with the 10-s dataset, images were partitioned into an 80% training subset and a 20% validation subset for model development.

### Model Training and Development

All models were trained on a Kent State University server running Ubuntu Linux 22.04.5 LTS, equipped with a single NVIDIA A40 GPU, 32 GB of DDR5 RAM, and 32 CPU cores, using PyTorch 2.8.0 + cu128, cuDNN 91002, Ultralytics YOLO 8.3.7, NVIDIA driver 575.64.03, and CUDA 12.9 runtime. The object-detection model was the pretrained YOLOv8x from the Ultralytics library. Images were resized to 1,280 × 1,280 pixels during both training and inference. While the default YOLOv8 input size is 640 × 640, we used the higher resolution to better capture fine morphological details of beetle species in our controlled laboratory environment (Ultralytics 2023).

For the 10-s subset, the model’s final head layer was reinitialized to match the number of classes (3, one for each insect species) in the custom dataset. The second model, trained on the larger 1-min dataset, was initialized with the 10-s model’s best checkpoint (best.pt) to leverage learned features and speed convergence. This transfer learning approach leveraged features learned from the 10-s dataset to accelerate training on the larger, more complex dataset, and no further architectural changes were required.

To improve model robustness and generalization, default data augmentation techniques were applied during training (Jocher et al. 2023). These techniques, such as hue, saturation, value adjustments, increased tolerance to lighting variations, while mosaic augmentation helped the model learn from varied spatial contexts.

The model was trained for 100 epochs using Ultralytics’ automatic optimizer selection. Under this setting, YOLOv8 selects stochastic gradient descent (SGD) for longer runs (>10,000 iterations) with an initial learning rate lr0 = 0.01 and momentum = 0.9, and selects AdamW with a class_**‑**_dependent initial learning rate for shorter runs. In our configuration (total iterations >10,000), “auto” resolved to SGD with lr0 = 0.01. Early stopping terminated training if validation performance did not improve for 10 consecutive epochs (Jocher et al. 2023).

### Model Evaluation

Model performance was evaluated using Ultralytics’ default validation metrics (Appendix S1—see online supplementary material). We did not perform any hypothesis-testing statistical methods here because the model performance metrics provide a direct, standard evaluation of classification and detection performance. By default, YOLO saves the best checkpoint based on mAP@0.5 to 0.95. mAP@0.5 to 0.95 summarizes how accurately the model detects objects by measuring how well predicted bounding boxes match the annotated ground-truth boxes across a range of overlap thresholds, with higher values indicating better detection performance. Accordingly, the final model (best.pt) corresponds to the epoch with the highest mAP@0.5 to 0.95. We additionally report mAP@0.5, precision–recall curves, F1-score, and a confusion matrix for completeness; the F1-score reflects the tradeoff between missed detections and false positives, while the confusion matrix summarizes correct classifications and common misclassification patterns across classes (Jocher et al. 2023).

## Results

### 10-s Set

**Fig. 2.**
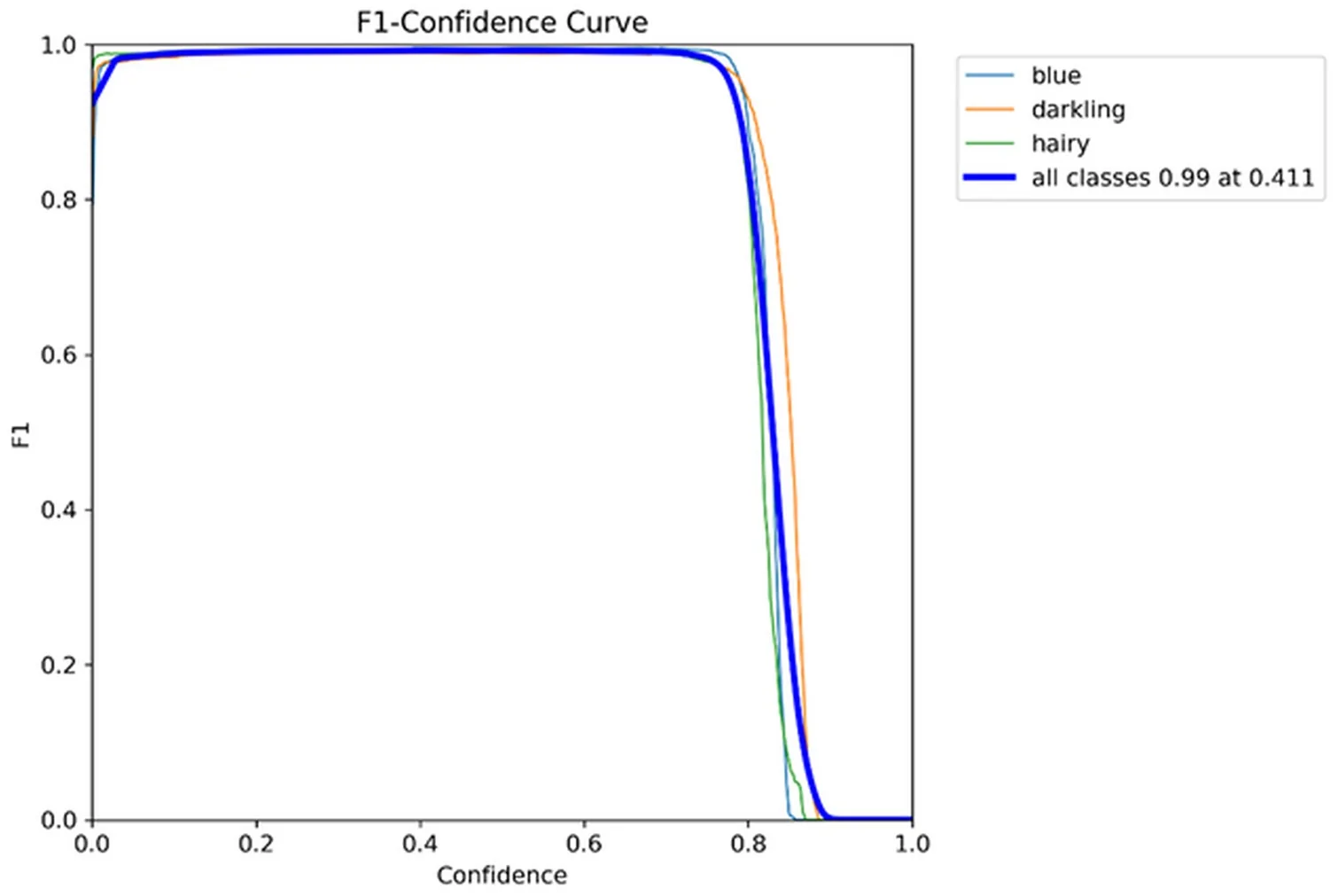
F1-confidence curve for the calibration model displaying F1-score as a function of confidence threshold for each beetle class and the overall model. The F1-score represents the harmonic mean of precision and recall. Optimal performance for this dataset was achieved at a confidence threshold of 0.411, yielding an F1-score of 0.99.

In total, we had 10 unique species-count configurations and 6,893 images overall, including 1,876 background images, the majority of which came from the standard tote runs. The object-detection models were broadly able to identify beetles present in photos. The 10-s set contained a total of 9,887 annotations: 3,759 blue death feigning beetle annotations, 2,822 darkling beetle annotations, and 3,306 hairy robot beetle annotations. The model demonstrated high performance across all evaluated metrics. After 31 epochs of training, the model achieved an overall F1-score of 0.99 at a confidence threshold of 0.41, with class-specific curves remaining near 1.0 across the tested range (Fig. 2). For the validation dataset, the mean average precision at 0.5 Intersection over Union (IoU) (mAP@0.5) was 0.994, and the mean average precision across thresholds from 0.5 to 0.95 (mAP@0.5:0.95) was 0.74.

**Fig. 3.**
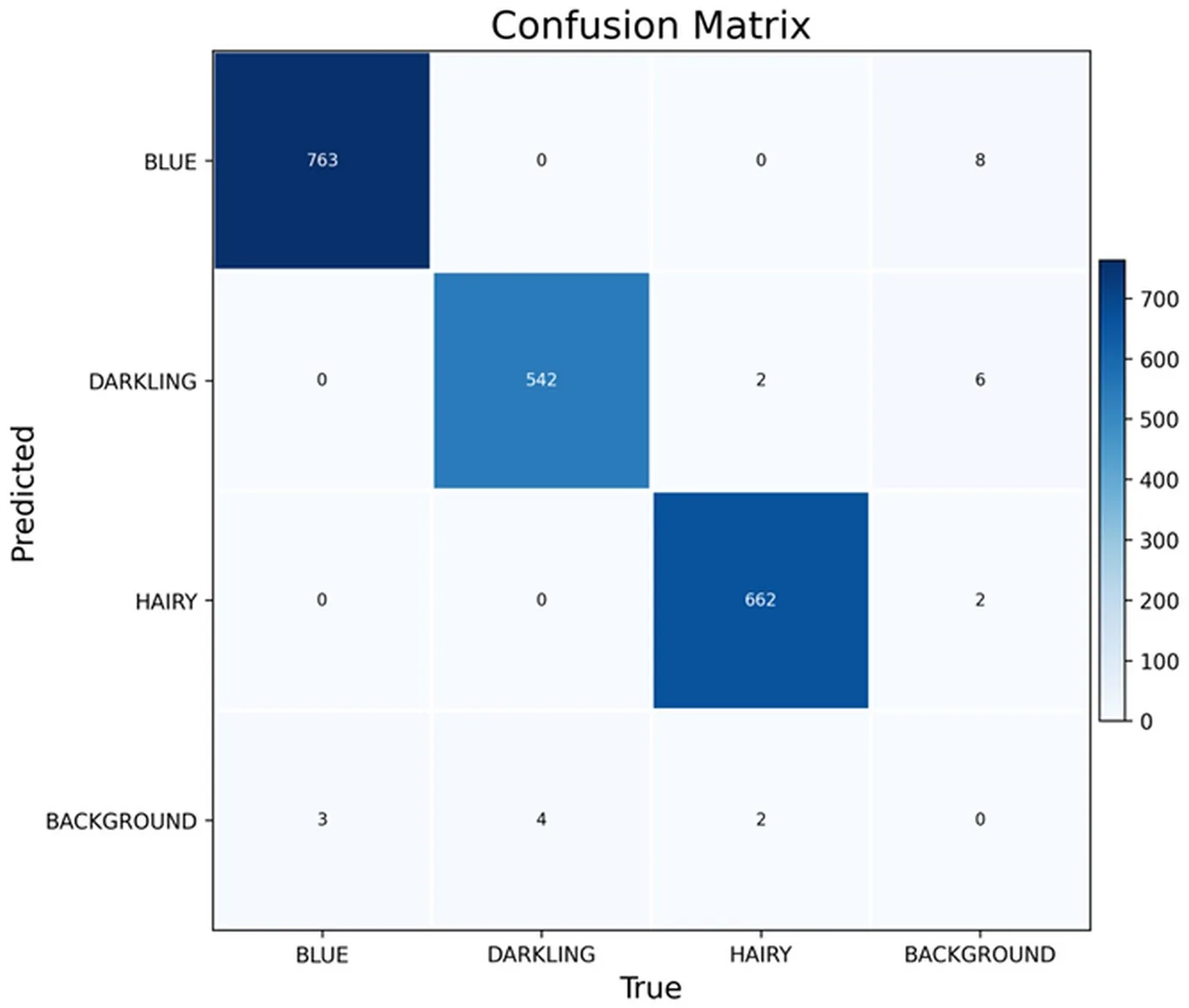
Confusion matrix for the calibration model showing the number of predictions for each true class-predicted class combination. A confusion matrix compares the true class (rows) to the predicted class (columns). A perfect model would have all counts on the diagonal, while off-diagonal cells indicate misclassification.

A confusion matrix for the 10-s model (Fig. 3) compares the true class (rows) to the predicted class (columns). A perfect model places counts on the diagonal, and off-diagonal cells indicate misclassifications. For each species, we had very low misclassification. Blue death feigning beetles were misclassified in 0.4% of images, darkling beetles were misclassified in 0.7% of images, and hairy robot beetles were misclassified in 0.6% of images. Indeed, most counts lie on the diagonal of the confusion matrix, indicating species were classified correctly (Fig. 3).

### 1-min Set

**Fig. 4.**
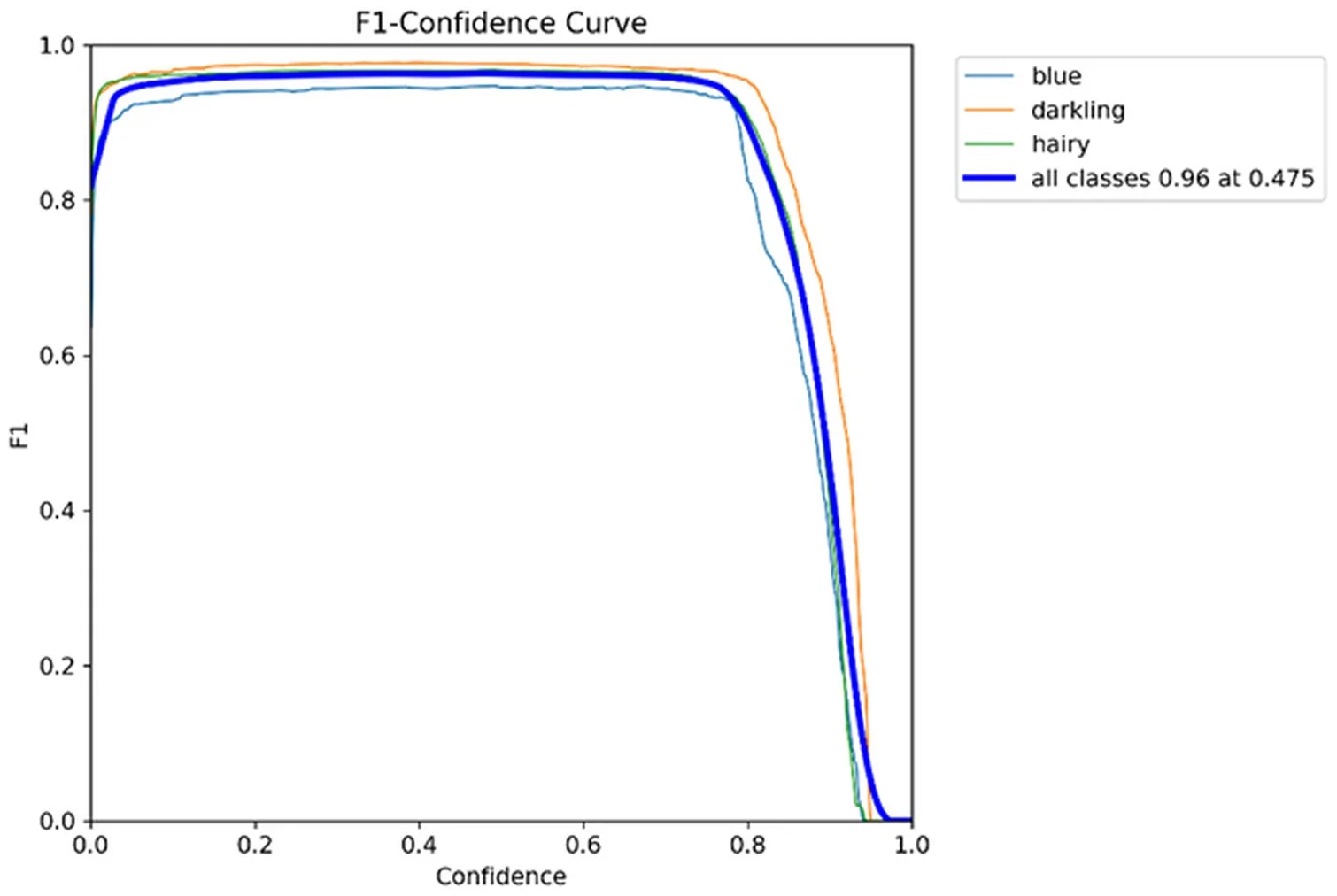
The F1-confidence curve for the 1-min model displaying F1-score as a function of confidence threshold for each beetle class and the overall model. The F1-score represents the harmonic mean of precision and recall. Optimal performance for this dataset was achieved at a confidence threshold of 0.475, yielding an F1-score of 0.96.

For the 1-min interval dataset, we collected 40,060 images. The model was trained for 100 epochs and demonstrated consistent improvement across training with the best model achieved at epoch 99. The dataset contained 16,008 total annotations: 2,778 blue death feigning beetle, 7,958 darkling beetle, and 5,272 hairy robot beetle. The model achieved an F1-score of 0.98, with class-specific curves remaining above 0.95 across the full confidence range (Fig. 4). For the validation dataset, the mean average precision at 0.5 IoU (mAP@0.5) reached 0.98, and the mean average precision across thresholds from 0.5 to 0.95 (mAP@0.5:0.95) was 0.84.

**Fig. 5.**
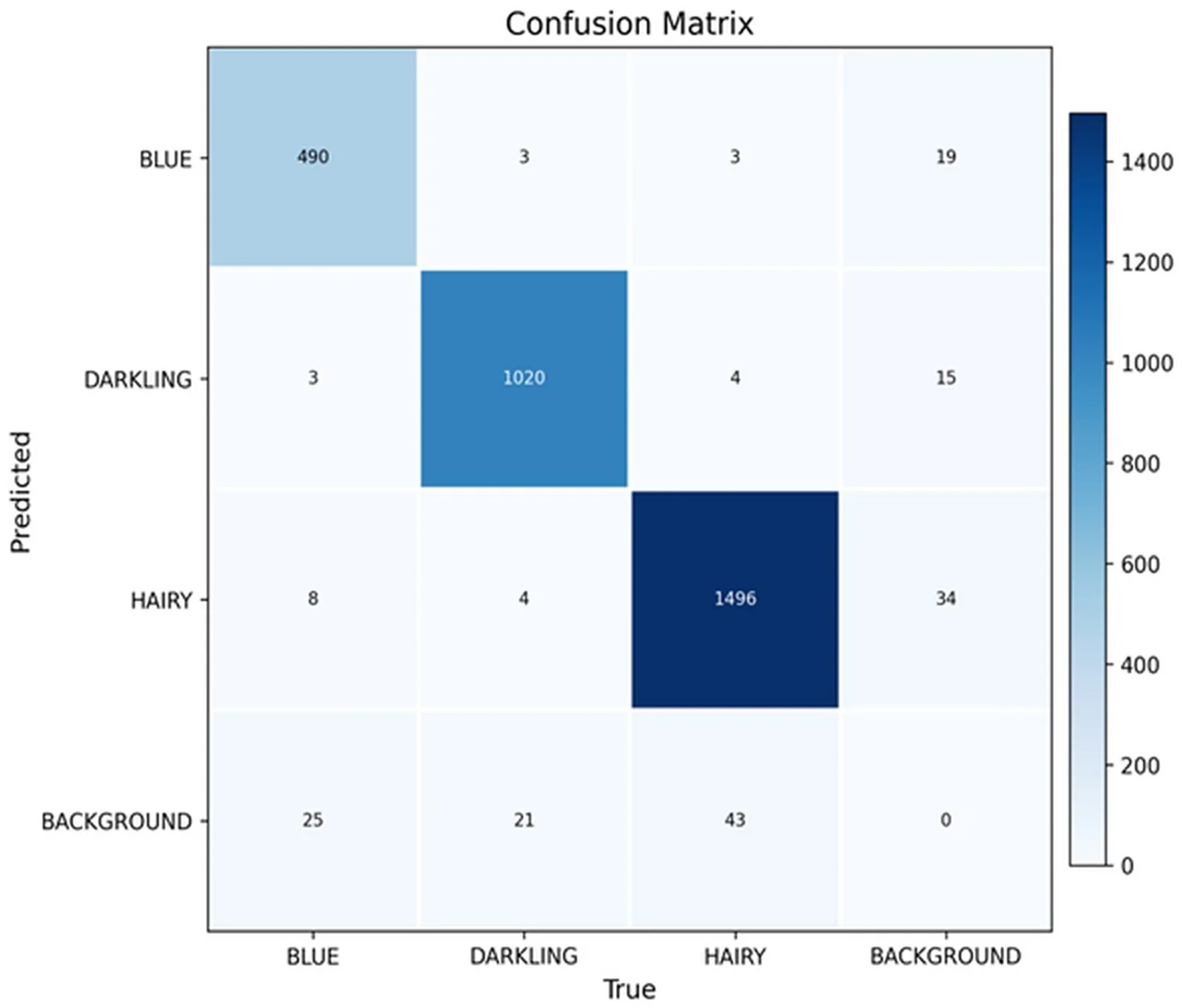
Confusion matrix for the 1-min model showing the number of predictions for each true class-predicted class combination. A confusion matrix compares the true class (rows) to the predicted class (columns). A perfect model would have all counts on the diagonal, while off-diagonal cells indicate misclassification.

A confusion matrix for the 1-min model compares the true class to the predicted class (Fig. 5). For each species, we had very low misclassification, although the percentage of misclassified beetles was higher in this set relative to the 10-s set; 6.8% of blue death feigning beetle images were misclassified, while 2.7% and 3.3% of darkling and hairy robot beetle images were misclassified, respectively. Despite this higher rate of misclassification, the majority of detections were correctly classified by our model (Fig. 5), indicating high performance.

## Discussion

Altogether, we find that our camera setup and data processing and image annotation workflow functioned effectively in a laboratory setting. This work demonstrated a low-cost method to identify ground-dwelling insects using commercially available hardware and an open-source data processing workflow. Ultimately, we expect this work can facilitate more accessible and streamlined insect biodiversity monitoring. Further, a useful aspect of the workflow described here is the modularity. We combined varied aspects of hardware and software to develop our workflow, which can make adoption and adaptation of this approach by other researchers more accessible.

Both the 10-s YOLOv8x model (Fig. 2) and the 1-min YOLOv8x model (Fig. 4) performed well across species. Under controlled conditions, the models were able to correctly identify practically all specimens with few exceptions. Given the limited classes and mostly static background, strong performance with a high-capacity model is expected. Prior work indicates that in similarly controlled settings, lighter detectors can offer comparable accuracy at much lower computing and power cost (Ahmad et al. 2022). However, we selected YOLOv8x to ease the transition to field conditions, where dynamic backgrounds and variable insect sizes demand more capacity. This necessity for a more computationally heavy model was demonstrated with the modest drop in performance with the 1-min model. The 1-min dataset had greater occlusion and more variation in beetle orientation and appearance relative to the constrained calibration sets.

Across classes, the lowest performance occurred for the hairy robot beetle, primarily due to background confusion, its tendency to remain beneath the ornamental vegetation in the frame, and the combined effects of dark coloration, shadowing, and small body size. This pattern is consistent with known small-object and low-contrast challenges in insect imaging (eg Bjerge et al. 2023, Ma et al. 2024, Hakim et al. 2025), which could be mitigated by increasing feature resolution/capacity and by tiling or slicing high-resolution images to enlarge the effective object size (Fotouhi et al. 2024). In addition, modifying the detector architecture can boost performance on tiny insects (Yue et al. 2024, Liu et al. 2025). Alternatively, if computational or architectural modifications prove insufficient, the Wingscapes TimelapseCam Pro can capture images at closer distances (as close as 15 cm), which would increase object size in the frame at the cost of a reduced field of view and potentially lower capture rates.

In our laboratory testing at 38 cm, the camera consistently produced high-quality images without frequent battery replacement, which suggests it may be suitable for deployment in field-settings. Indeed, the ability to produce high-resolution, close-focus images with support for interval capture and nighttime operation is essential for utility in heterogeneous field environments. Nighttime operation is especially relevant when monitoring many insect indicator groups such as ground beetles (Coleoptera: Carabidae), since many species are primarily active at night (Rainio and Niemelä 2003, Naqvi et al. 2022). This was a limitation in study design because the built-in flash was not required under indoor lighting, thus its effectiveness for 24-h field deployment remains to be validated.

### Future Directions and Application

The methodological workflow described herein establishes a foundation for application and testing of this approach in field-based biodiversity studies. Indeed, the next step is to apply this lab-tested approach to field conditions. To our knowledge, there is no published, ground-oriented camera-trap workflow for ground-dwelling arthropods in which cameras image the substrate itself, and field images are automatically processed by an object detector. In addition to collecting biodiversity data, ground-oriented cameras that are continuously deployed could provide more detailed data on naturally occurring insect populations by documenting diel activity patterns, in addition to occurrence and abundance data. Moreover, this tool could be applied to monitor the spread and occurrence of invasive insects. There is however a need to refine current statistical methods for analyzing camera-trap images in order to provide robust estimates of abundance, richness, and occurrence patterns. Documenting activity patterns of insects represents a fundamentally different observation process than traditional insect trapping approaches (Boyd et al. 2023, 2024), thus analysis of camera-trap data necessitates careful consideration and planning. For example, field data using this method may accrue double counts of individuals repeatedly moving through the camera area, thus hindering estimates of abundance or diversity. In entomology, efforts to limit double counts have mostly relied on postprocessing. For example, merging track fragments across adjacent frames with tracking-by-detection (Bjerge et al. 2021, Sittinger et al. 2024). Outside entomology, more extensive approaches are used, such as calibrating with mark—recapture or mark—resight of physically marked animals (Forti et al. 2022) and applying hierarchical models that account for imperfect detection (Zipkin et al. 2017, Nakashima et al. 2022, Harris et al. 2024). Accounting for double counts in camera-trap data is an area of active research (Gilbert et al. 2021) and is essential to developing an analytic pipeline that accurately estimates relevant biodiversity metrics from camera-trap data.

To help calibrate cameras in the field, it may be useful to deploy colocated pitfall traps in tandem with camera traps for a subset of the time cameras are deployed. This can be used to verify species detected on cameras and provide an estimate of the species available for detection in the camera trap. However, since pitfall traps destructively sample (ie sample without replacement), this approach should be used sparingly to retain the benefit of nonextractive sampling from the camera traps. Combining traditional insect collection data with camera-trap data may be a powerful way to gain robust inference concerning occurrence, abundance, and richness patterns among insects.

## Supplementary Material

ieag091_Supplementary_Data

## Data Availability

All data and code used in this project are available at the following repository: https://github.com/Rjb4444/Machine-Learning-Workflows-Detect-Ground-Dwelling-Arthropods-Against-Heterogenous-Backgrounds. The graphical abstract was created in BioRender (https://BioRender.com).

## References

[ieag091-B1] Abadi M , Barham P, Chen J, et al 2016. *TensorFlow: A system for large-scale machine learning*. arXiv. https://arxiv.org/abs/1605.08695

[ieag091-B2] Ahmad I , Yang Y, Yue Y, et al 2022. Deep learning based detector YOLOv5 for identifying insect pests. Appl. Sci. 12:10167. 10.3390/app121910167

[ieag091-B3] Alison J , Alexander JM, Diaz Zeugin N, et al 2022. Moths complement bumblebee pollination of red clover: a case for day-and-night insect surveillance. Biol. Lett. 18:20220187. 10.1098/rsbl.2022.0187 35857892 PMC9277237

[ieag091-B5] Bjerge K , Alison J, Dyrmann M, et al 2023. Accurate detection and identification of insects from camera trap images with deep learning. PLOS Sustain. Transform. 2:e0000051. 10.1371/journal.pstr.0000051

[ieag091-B6] Bjerge K , Frigaard CE, Karstoft H. 2023. Object detection of small insects in time-lapse camera recordings. Sensors (Basel). 23:7242. 10.3390/s23167242 37631778 PMC10459366

[ieag091-B7] Bjerge K , Karstoft H, Mann HMR, et al 2024. A deep learning pipeline for time-lapse camera monitoring of insects and their floral environments. Ecol. Inform. 84:102861. 10.1016/j.ecoinf.2024.102861

[ieag091-B8] Bjerge K , Nielsen JB, Sepstrup MV, et al 2021. An automated light trap to monitor moths (Lepidoptera) using computer vision-based tracking and deep learning. Sensors (Basel). 21:343. 10.3390/s21020343 33419136 PMC7825571

[ieag091-B10] Boyd RJ , Powney GD, Pescott OL. 2023. We need to talk about nonprobability samples. Trends Ecol. Evol. 38:521–531. 10.1016/j.tree.2023.01.001 36775795

[ieag091-B11] Boyd RJ , Stewart GB, Pescott OL. 2024. Descriptive inference using large, unrepresentative nonprobability samples: an introduction for ecologists. Ecology. 105:e4214. 10.1002/ecy.4214 38088061 PMC10929663

[ieag091-B12] Burton AC , Neilson E, Moreira D, et al 2015. REVIEW: wildlife camera trapping: a review and recommendations for linking surveys to ecological processes. J. Appl. Ecol. 52:675–685. 10.1111/1365-2664.12432

[ieag091-B13] East A , Campolongo EG, Meyers L, et al 2025. Optimizing image capture for computer vision‐powered taxonomic identification and trait recognition of biodiversity specimens. Methods Ecol. Evol. 16:2260–2275. 10.1111/2041-210x.70140

[ieag091-B14] Engel MS , Ceríaco LMP, Daniel GM, et al 2021. The taxonomic impediment: a shortage of taxonomists, not the lack of technical approaches. Zool. J. Linnean Soc. 193:381–387. 10.1093/zoolinnean/zlab072

[ieag091-B15] Fischer B , Larson BMH. 2019. Collecting insects to conserve them: a call for ethical caution. Insect Conserv. Divers. 12:173–182. 10.1111/icad.12344

[ieag091-B16] Forti A , Partel P, Orsingher MJ, et al 2022. A comparison of capture-mark-recapture and camera-based mark-resight to estimate abundance of Alpine marmot (*Marmota marmota*). J. Vertebr. Biol. 71:22023.1-11. 10.25225/jvb.22023

[ieag091-B17] Fotouhi F , Menke K, Prestholt A, et al 2024. Persistent monitoring of insect-pests on sticky traps through hierarchical transfer learning and slicing-aided hyper inference. Front. Plant Sci. 15:1484587. 10.3389/fpls.2024.1484587 39649808 PMC11624506

[ieag091-B18] Gilbert NA , Clare JDJ, Stenglein JL, et al 2021. Abundance estimation of unmarked animals based on camera-trap data. Conserv. Biol. 35:88–100. 10.1111/cobi.13517 32297655

[ieag091-B19] Hakim A , Srivastava AK, Hamza A, et al 2025. Yolo-pest: an optimized YoloV8x for detection of small insect pests using smart traps. Sci. Rep. 15:14029. 10.1038/s41598-025-97825-3 40269001 PMC12019348

[ieag091-B20] Harris GM , Stewart DR, Butler MJ, et al 2024. N-mixture models with camera trap imagery produce accurate abundance estimates of ungulates. Sci. Rep. 14:31421. 10.1038/s41598-024-83011-4 39733085 PMC11682081

[ieag091-B21] Hoekman D , LeVan KE, Gibson C, et al 2017. Design for ground beetle abundance and diversity sampling within the National Ecological Observatory Network. Ecosphere. 8:e01744. 10.1002/ecs2.1744

[ieag091-B22] Høye TT , Ärje J, Bjerge K, et al 2021. Deep learning and computer vision will transform entomology. Proc. Natl. Acad. Sci. U. S. A. 118:e2002545117. 10.1073/pnas.2002545117 33431561 PMC7812775

[ieag091-B23] Høye TT , Montagna M, Oteman B, et al 2025. Emerging technologies for pollinator monitoring. Curr. Opin. Insect Sci. 69:101367. 10.1016/j.cois.2025.101367 40086507

[ieag091-B24] Huang Y , Liu Z, Zhao H, et al 2025. YOLO-YSTs: an improved YOLOv10n-based method for real-time field pest detection. Agronomy. 15:575. 10.3390/agronomy15030575

[ieag091-B25] Jocher G , Chaurasia A, Qiu J. 2023. Yolov8_ultralytics (Version 8.0.0) [Computer software]. https://docs.ultralytics.com/

[ieag091-B26] Liu J , Li Y, Wang L, et al 2025. GIWT-YOLO: an efficient multi-scale framework for real-time Scolytinae pests detection. Front. Insect Sci. 5:1635439. 10.3389/finsc.2025.1635439 41079258 PMC12511143

[ieag091-B27] Lv J , Li W, Fan M, et al 2022. Detecting pests from light-trapping images based on improved YOLOv3 model and instance augmentation. Front. Plant Sci. 13:939498. 10.3389/fpls.2022.939498 35873992 PMC9301456

[ieag091-B28] Ma Y , Wei Y, Ma M, et al 2024. DCP-YOLOv7x: Improved pest detection method for low-quality cotton image. Front. Plant Sci. 15:1501043. 10.3389/fpls.2024.1501043 39748823 PMC11693457

[ieag091-B30] Nachman MW , Beckman EJ, Bowie RC, et al 2023. Specimen collection is essential for modern science. PLoS Biol. 21:e3002318. 10.1371/journal.pbio.3002318 37992027 PMC10664955

[ieag091-B31] Nakashima Y , Hongo S, Mizuno K, et al 2022. Double-observer approach with camera traps can correct imperfect detection and improve the accuracy of density estimation of unmarked animal populations. Sci. Rep. 12:2011. 10.1038/s41598-022-05853-0 35132116 PMC8821540

[ieag091-B32] Naqvi Q , Wolff PJ, Molano-Flores B, et al 2022. Camera traps are an effective tool for monitoring insect–plant interactions. Ecol. Evol. 12:e8962. 10.1002/ece3.8962 35784070 PMC9163375

[ieag091-B33] Norouzzadeh MS , Nguyen A, Kosmala M, et al 2018. Automatically identifying, counting, and describing wild animals in camera-trap images with deep learning. Proc. Natl. Acad. Sci. U. S. A. 115:E5716–E5725. 10.1073/pnas.1719367115 29871948 PMC6016780

[ieag091-B34] Paszke A , Gross S, Massa F, et al 2019. PyTorch: an imperative style, high-performance deep learning library. In: Wallach HM, Larochelle H, Beygelzimer A, d’Alché Buc F, Fox EB, Garnett R, editors. NeurIPS. p. 8024–8035. http://dblp.uni-trier.de/db/conf/nips/nips2019.html#PaszkeGMLBCKLGA19.

[ieag091-B8250840] Pereira . Fall Detection for Industrial Setups Using YOLOv8 Variants. arXiv 2024. https://arxiv.org/abs/2408.04605

[ieag091-B35] Preti M , Verheggen F, Angeli S. 2021. Insect pest monitoring with camera-equipped traps: strengths and limitations. J. Pest Sci. 94:203–217. 10.1007/s10340-020-01309-4

[ieag091-B36] Rainio J , Niemelä J. 2003. Ground beetles (Coleoptera: Carabidae) as bioindicators. Biodivers. Conserv. 12:487–506. 10.1023/A : 1022412617568

[ieag091-B37] Redmon J , Divvala S, Girshick R, et al 2016. You only look once: unified, real-time object detection. In: 2016 IEEE Conference on Computer Vision and Pattern Recognition (CVPR). p. 779–788. 10.1109/CVPR.2016.91

[ieag091-B38] Roy DB , Alison J, August TA, et al 2024. Towards a standardized framework for AI-assisted, image-based monitoring of nocturnal insects. Philos. Trans. R. Soc. Lond. B Biol. Sci. 379:20230108. 10.1098/rstb.2023.0108 38705190 PMC11070254

[ieag091-B39] Seimandi-Corda G , Hood T, Cook SM. 2025. Understanding insect predator–prey interactions using camera trapping: a review of current research and perspectives. Agri. Forest Entomol. 27:67–80. 10.1111/afe.12646

[ieag091-B40] Seimandi-Corda G , Hood T, Hampson M, et al 2024. Identifying insect predators using camera traps reveal unexpected predator communities in oilseed rape fields. Biol. Control 198:105636. 10.1016/j.biocontrol.2024.105636

[ieag091-B41] Sittinger M , Uhler J, Pink M, et al 2024. Insect detect: an open-source DIY camera trap for automated insect monitoring. PLoS One. 19:e0295474. 10.1371/journal.pone.0295474 38568922 PMC10990185

[ieag091-B42] Spiesman BJ , Gratton C, Hatfield RG, et al 2021. Assessing the potential for deep learning and computer vision to identify bumble bee species from images. Sci. Rep. 11:7580. 10.1038/s41598-021-87210-1 33828196 PMC8027374

[ieag091-B43] Stark T , Ştefan V, Wurm M, et al 2023. YOLO object detection models can locate and classify broad groups of flower-visiting arthropods in images. Sci. Rep. 13:16364. 10.1038/s41598-023-43482-3 37773202 PMC10541899

[ieag091-B44] Szeliski R. 2022. Computer vision: algorithms and applications. 2nd ed.

[ieag091-B8668638] Tkachenko M , Malyuk M, Holmanyuk A, et al. *Label Studio: Data labelling Software*. Human Signal. 2025. https://github.com/HumanSignal/label-studio

[ieag091-B46] Valan M , Makonyi K, Maki A, et al 2019. Automated taxonomic identification of insects with expert-level accuracy using effective feature transfer from convolutional networks. Syst. Biol. 68:876–895. 10.1093/sysbio/syz014 30825372 PMC6802574

[ieag091-B47] van Klink R , August T, Bas Y, et al 2022. Emerging technologies revolutionise insect ecology and monitoring. Trends Ecol. Evol. 37:872–885. 10.1016/j.tree.2022.06.001. 35811172

[ieag091-B48] Venverloo T , Duarte F. 2024. Towards real-time monitoring of insect species populations. Sci. Rep. 14:18727. 10.1038/s41598-024-68502-8 39134595 PMC11319484

[ieag091-B49] Wagner DL. 2020. Insect declines in the Anthropocene. Annu. Rev. Entomol. 65:457–480. 10.1146/annurev-ento-011019-025151 31610138

[ieag091-B50] Wittmann K , Gamal Ibrahim M, Straw AD, et al 2024. Monitoring fast-moving animals—building a customized camera system and evaluation toolset. Methods Ecol. Evol. 15:836–842. 10.1111/2041-210X.14322

[ieag091-B51] Yue G , Liu Y, Niu T, et al 2024. GLU-YOLOv8: an improved pest and disease target detection algorithm based on YOLOv8. Forests. 15:1486. 10.3390/f15091486

[ieag091-B52] Zarboubi M , Bellout A, Chabaa S, et al 2026. Enhancing integrated pest management with IoT and YOLO-Evo: a smart, low-cost monitoring system for sustainable apple farming. Results Eng. 29:108850. 10.1016/j.rineng.2025.108850

[ieag091-B53] Zipkin EF , Rossman S, Yackulic CB, et al 2017. Integrating count and detection–nondetection data to model population dynamics. Ecology. 98:1640–1650. 10.1002/ecy.1831 28369775

