# Supplementary material for: Machine learning workflows detect ground-dwelling arthropods against heterogenous backgrounds
Source: J Insect Sci. 2026 Sep 1;26(5):ieag091. doi: 10.1093/jisesa/ieag091 (PMC13533566; doi:10.1093/jisesa/ieag091)
Supplement: Supplementary file 1 [file ieag091_supplementary_data.docx]

**Supplementary material**

**Appendix S1: Formula for model performance metrics**

Model performance was evaluated using precision (P), recall (R), average precision (AP), mean average precision (mAP), and the F₁-score (F1) as follows:

          (1)

           (2)

where TP, FP, and FN represent true positives, false positives, and false negatives, respectively.
 Precision (P) measures the proportion of correctly identified positive detections out of all detections made by the model, while recall (R) measures the proportion of correctly identified positives out of all actual positive instances.

(3)

Average precision (AP) summarizes the area under the precision–recall curve for a single class, integrating precision over recall.

(4)

Mean average precision (mAP) is the mean of AP values across all N classes, providing an overall measure of detection performance.

(5)

The F₁-score combines precision and recall into a single harmonic mean, with higher values indicating a stronger balance between detection accuracy and completeness. For F1 calculations the optimal thresholds calculated using scikit were used.
